# Supplementary material for: Health Effects of Drinking Water Produced from Deep Sea Water: A Randomized Double-Blind Controlled Trial
Source: Nutrients. 2022 Jan 28;14(3):581. doi: 10.3390/nu14030581 (PMC8839038; doi:10.3390/nu14030581)
Supplement: Supplementary file 1 [file nutrients-14-00581-s001.zip › nutrients-1526190-supplementary.pdf]

Supplementary Table S1. Nutrition facts of Dydo-MIU, hardness, 88 (100mL).

Composition (mg)			
Sodium (1.4)	Calcium (0.6)	Magnesium (1.8)	Potassium (0.9)
Total fat (0)	Protein (0)	Sugar (0)	Carbohydrate (0)
Calories (0)			

Ca:Mg = 1:3
